# Subcutaneous Myeloma Deposit in the Region of an Arteriovenous Fistula

**DOI:** 10.4274/tjh.2016.0335

**Published:** 2017-08-02

**Authors:** Petar Djuric, Aleksandar Jankovic, Zoran Milojevic, Katarina Markovic, Slavisa Sekulic, Milan Pantelic, Jelena Tosic Dragovic, Ana Bulatovic, Nada Dimkovic

**Affiliations:** 1 Zvezdara University Medical Center, Department of Nephrology, Belgrade, Serbia; 2 Zvezdara University Medical Center, Department of Hematology, Belgrade, Serbia; 3 Zvezdara University Medical Center, Department of Surgery, Belgrade, Serbia; 4 Zvezdara University Medical Center, Department of Radiology, Belgrade, Serbia; 5 Belgrade of University Faculty of Medicine, Department of Nephrology, Belgrade, Serbia

**Keywords:** Distal arteriovenous fistula, Multislice computer tomography, Hemodialysis

A 78-year-old male was hospitalized in October 2013 due to renal failure. Soon thereafter, light-chain deposition disease was confirmed (lambda type DSSS IIIA, ISS III). A high-dose DEXA protocol was introduced and he received 11 protocols during the following 12 months. In October 2014 he commenced maintenance hemodialysis (HD) via a distal arteriovenous fistula (AVF). In March 2015 he noticed swelling of the fistula region. Although the AVF was functional, local findings on the skin deteriorated within 1 month (Figure 1). Multislice computer tomography demonstrated highly vascularized tumor-like changes originating from the AVF (Figure 2). The patient underwent aspiration biopsy of the skin and more than 10% lymphoplasmacytic cells were found by microscopy. The finding was confirmed by histology (Figure 3). A PET scan was not available. At that time, he was very frail and no specific therapy was recommended by the hematologist. The patient died within 3 weeks.

Presentation of extramedullary subcutaneous light-chain deposition surrounding an AVF may be a potential link between light-chain deposition disease and augmented circulation, thus giving a preferential site for tumor growth [1]. Hematogenous spread (metastasis) to the AVF region is plausible considering the intact adjacent bone and repeated trauma of the multiple cannulation of the AVF. Such repeated trauma gives a good environment for tumor seeding. Our conclusion is that patients with light-chain deposition disease and end-stage renal disease may be considered for peritoneal dialysis instead of HD.

## Figures and Tables

**Figure 1:**
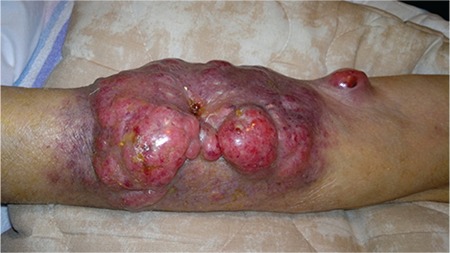
Local finding on the skin of the left forearm: note that the entire circumference of the forearm was affected by tumor-like changes.

**Figure 2:**
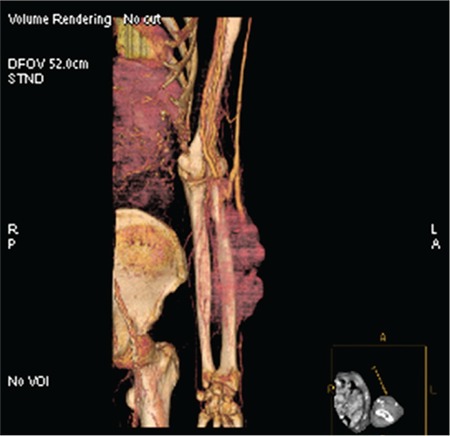
Multislice computer tomography angiography of the arteriovenous fistula.

**Figure 3:**
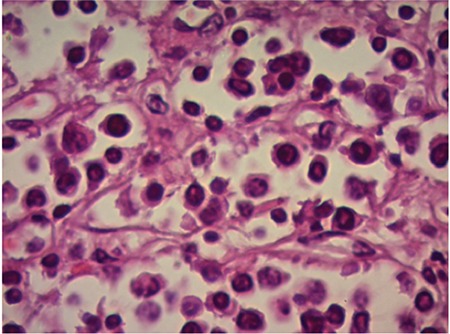
Histology of the affected skin: multiple lymphoplasmacytic cells.
